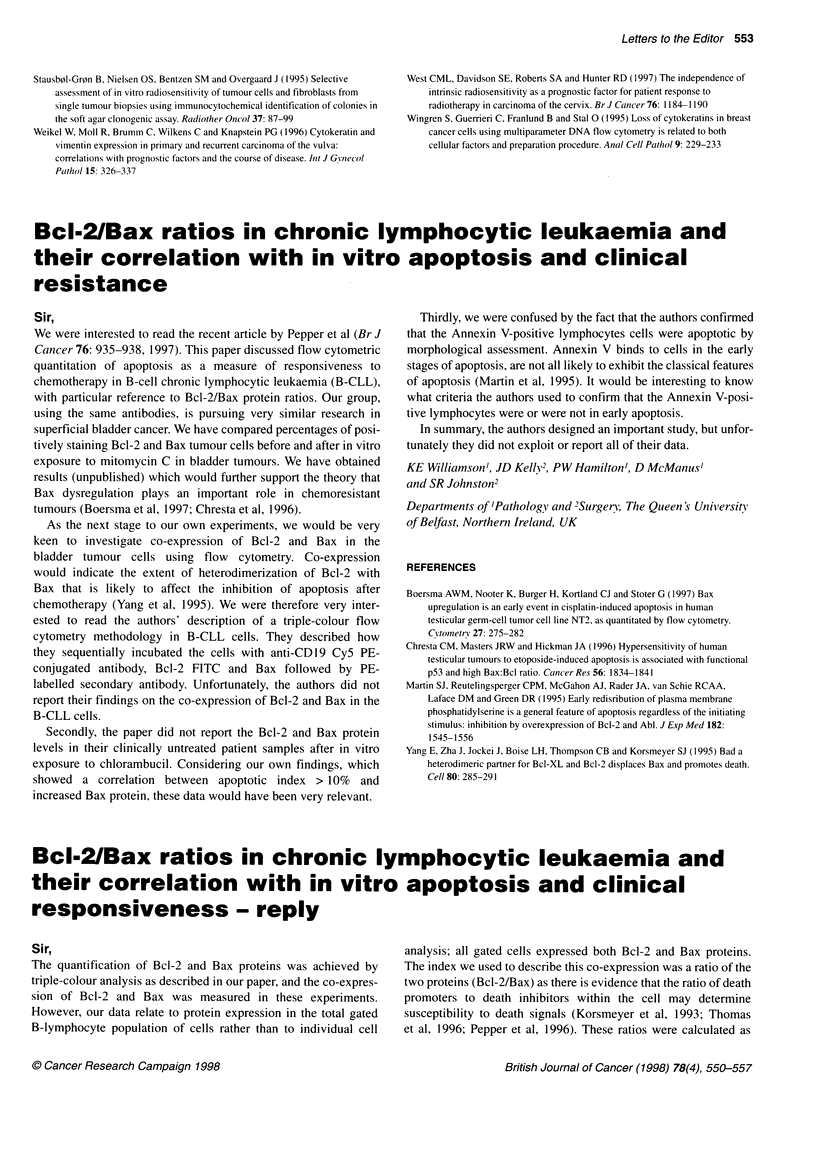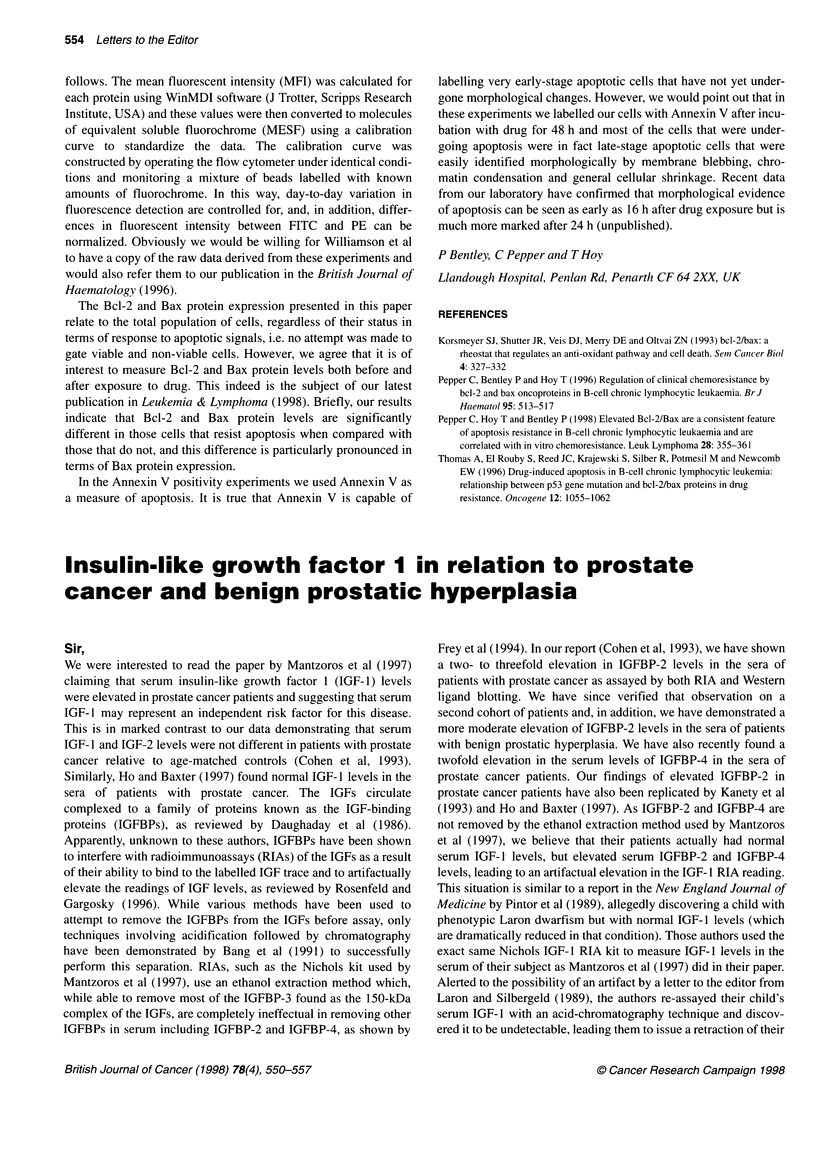# Bcl-2/Bax ratios in chronic lymphocytic leukaemia and their correlation with in vitro apoptosis and clinical responsiveness - reply

**Published:** 1998-08

**Authors:** P Bentley, C Pepper, T Hoy


### Bcl-2/Bax ratios in chronic lymphocytic leukaemia and their correlation with in vitro apoptosis and clinical responsiveness – reply

**Sir,**

The quantification of Bcl-2 and Bax proteins was achieved by triple-colour analysis as described in our paper, and the co-expression of Bcl-2 and Bax was measured in these experiments. However, our data relate to protein expression in the total gated B-lymphocyte population of cells rather than to individual cell analysis; all gated cells expressed both Bcl-2 and Bax proteins. The index we used to describe this co-expression was a ratio of the two proteins (Bcl-2/Bax) as there is evidence that the ratio of death promoters to death inhibitors within the cell may determine susceptibility to death signals (Korsmeyer et al, 1993; Thomas et al, 1996; Pepper et al, 1996). These ratios were calculated as

© Cancer Research Campaign 1998

follows. The mean fluorescent intensity (MFI) was calculated for each protein using WinMDI software (J Trotter, Scripps Research Institute, USA) and these values were then converted to molecules of equivalent soluble fluorochrome (MESF) using a calibration curve to standardize the data. The calibration curve was constructed by operating the flow cytometer under identical conditions and monitoring a mixture of beads labelled with known amounts of fluorochrome. In this way, day-to-day variation in fluorescence detection are controlled for, and, in addition, differences in fluorescent intensity between FITC and PE can be normalized. Obviously we would be willing for Williamson et al to have a copy of the raw data derived from these experiments and would also refer them to our publication in the *British Journal of Haematology* (1996).

The Bcl-2 and Bax protein expression presented in this paper relate to the total population of cells, regardless of their status in terms of response to apoptotic signals, i.e. no attempt was made to gate viable and non-viable cells. However, we agree that it is of interest to measure Bcl-2 and Bax protein levels both before and after exposure to drug. This indeed is the subject of our latest publication in *Leukemia & Lymphoma* (1998). Briefly, our results indicate that Bcl-2 and Bax protein levels are significantly different in those cells that resist apoptosis when compared with those that do not, and this difference is particularly pronounced in terms of Bax protein expression.

In the Annexin V positivity experiments we used Annexin V as a measure of apoptosis. It is true that Annexin V is capable of labelling very early-stage apoptotic cells that have not yet undergone morphological changes. However, we would point out that in these experiments we labelled our cells with Annexin V after incubation with drug for 48 h and most of the cells that were undergoing apoptosis were in fact late-stage apoptotic cells that were easily identified morphologically by membrane blebbing, chromatin condensation and general cellular shrinkage. Recent data from our laboratory have confirmed that morphological evidence of apoptosis can be seen as early as 16 h after drug exposure but is much more marked after 24 h (unpublished).

*P Bentley, C Pepper and T Hoy*

*Llandough Hospital, Penlan Rd, Penarth CF 64 2XX, UK*

**REFERENCES**

Korsmeyer SJ, Shutter JR, Veis DJ, Merry DE and Oltvai ZN (1993) bcl-2/bax: a rheostat that regulates an anti-oxidant pathway and cell death. *Sem Cancer Biol* **4**: 327–332

Pepper C, Bentley P and Hoy T (1996) Regulation of clinical chemoresistance by bcl-2 and bax oncoproteins in B-cell chronic lymphocytic leukaemia. *Br J Haematol* **95**: 513–517

Pepper C, Hoy T and Bentley P (1998) Elevated Bcl-2/Bax are a consistent feature of apoptosis resistance in B-cell chronic lymphocytic leukaemia and are correlated with in vitro chemoresistance. Leuk Lymphoma **28**: 355–361

Thomas A, El Rouby S, Reed JC, Krajewski S, Silber R, Potmesil M and Newcomb EW (1996) Drug-induced apoptosis in B-cell chronic lymphocytic leukemia: relationship between p53 gene mutation and bcl-2/bax proteins in drug resistance. *Oncogene* **12**: 1055–1062